# Pediatric Shock Across Acute Emergencies: Age Patterns, Etiologic Subtypes, and Bedside Clinical Indicators in a Single-Centre Cohort

**DOI:** 10.3390/children13030366

**Published:** 2026-03-04

**Authors:** Cristina Elena Singer, Ion Dorin Pluta, Ștefănița Bianca Vintilescu, Popescu Elena Madalina, George Alin Stoica, Renata-Maria Varut, Pirscoveanu Denisa Floriana Vasilica, Virginia Radulescu, Nuica Valentina Geanina, Denisa Preoteasa, Mocanu Andreea Gabriela, Carmen Sirbulet

**Affiliations:** 1 Department of Mother and Baby, University of Medicine and Pharmacy of Craiova, 200349 Craiova, Romania; cristina.singer@umfcv.ro (C.E.S.); bianca.vintilescu@umfcv.ro (Ș.B.V.); madalina.popescu@umfcv.ro (P.E.M.); 2 Faculty of Medical and Behavioral Sciences, Constantin Brâncuși University of Târgu Jiu, 210185 Târgu Jiu, Romania; dorin.pluta@e-ucb.ro; 3 Department of Pediatric Surgery, Faculty of Medicine, University of Medicine and Pharmacy of Craiova, 200349 Craiova, Romania; alin.stoica@umfcv.ro; 4 Research Methodology Department, Faculty of Pharmacy, University of Medicine and Pharmacy of Craiova, 200349 Craiova, Romania; 5 Department of Neurology, Faculty of Medicine, University of Medicine and Pharmacy of Craiova, 200349 Craiova, Romania; denisa.pirscoveanu@umfcv.ro; 6 Department of Statistics, University of Medicine and Pharmacy of Craiova, 200349 Craiova, Romania; virginia.radulescu@umfcv.ro; 7 Clinical Pharmacist, Vâlcea County Emergency Hospital, 240284 Râmnicu Vâlcea, Romania; geany_nuica@yahoo.com (N.V.G.); denisa_chiosa@yahoo.com (D.P.); 8 Department of Pharmaceutical Technology, Faculty of Pharmacy, University of Medicine and Pharmacy of Craiova, 200349 Craiova, Romania; gabriela.mocanu@umfcv.ro; 9 Discipline of Anatomy, Department of Anatomy, University of Medicine and Pharmacy of Craiova, 200349 Craiova, Romania; carmen.sirbulet@umfcv.ro

**Keywords:** pediatric shock, emergency, heart failure, meningitis, dehydration, sepsis, children

## Abstract

**Background/Objectives:** Pediatric shock is a final common pathway of cardiovascular failure across diverse emergencies, yet data from mixed emergency cohorts outside intensive care units remain limited. This study aimed to describe the distribution, etiologic subtypes, and clinical correlates of shock in children presenting within a diagnosis-based emergency cohort. **Methods**: A retrospective single-centre study was conducted in children aged 0–16 years presenting with selected acute pediatric emergencies, among whom cases with and without shock were compared. Shock was defined using documented diagnoses and compatible hemodynamic features, and multiple etiologic types of shock were analyzed, including hypovolemic, septic, cardiogenic, and anaphylactic shock. Demographic and diagnostic variables—age, length of stay, organ support, age strata, and selected comorbidities—and baseline clinical features were compared between children with and without shock using non-parametric and χ^2^/Fisher’s exact tests. **Results**: Within the prespecified diagnosis-based analytic cohort, 36/128 children (28.1%) met the study criteria for shock and occurred across all prespecified acute pediatric emergency groups, with the highest proportional burden in heart failure and meningitis; this proportion should not be interpreted as an emergency-department prevalence estimate. Children with shock were younger, with clustering in infants < 1 year and those aged 5–9 years, and tended to stay longer in hospital. Pre-existing cardiac disease, severe dehydration, and altered mental status/coma were more frequent among children with shock. Septic and cardiogenic shock required the most intensive organ support. **Conclusions**: In this pediatric emergency cohort, shock emerged as a clinically relevant and etiologically heterogeneous complication across diverse acute presentations, with a distinct age-related vulnerability pattern and consistent associations with readily identifiable bedside clinical features. Simple bedside information—particularly cardiac comorbidity, dehydration, and altered consciousness—may assist the early recognition of children with evolving circulatory failure and support closer monitoring and timely escalation of care. By focusing on a mixed emergency population outside the intensive care unit, this study provides a real-world clinical perspective that may help refine early bedside assessment and improve vigilance for shock in pediatric emergency departments.

## 1. Introduction

Shock is a life-threatening clinical syndrome of circulatory failure characterized by inadequate tissue perfusion of vital organs. In children, shock represents a final common pathway for multiple acute illnesses and remains a major contributor to pediatric morbidity and mortality worldwide. Septic shock, in particular, is a leading cause of childhood hospitalization and death, with global estimates reporting tens of millions of pediatric sepsis cases annually and approximately 2.9 million associated deaths, predominantly in low-resource settings [1,2]. Pediatric shock encompasses several etiologic mechanisms, including hypovolemic, distributive (most commonly septic or anaphylactic), cardiogenic, and obstructive shock [3]. In emergency settings, hypovolemic and septic shock predominate; for example, one pediatric emergency department cohort reported sepsis in 57% of shock cases, hypovolemia in 24%, other distributive causes in 14%, and cardiogenic shock in 5% [4].

The clinical presentation of pediatric shock varies considerably with age and developmental stage. Neonates and young infants are particularly vulnerable to septic shock, often presenting with subtle or non-specific manifestations such as hypothermia, lethargy, or poor feeding rather than overt fever. In contrast, toddlers and preschool-aged children more commonly develop hypovolemic shock due to severe dehydration associated with diarrheal disease, while older children and adolescents may present with distributive or traumatic hemorrhagic shock. Importantly, children exhibit strong age-dependent physiological compensation, frequently maintaining near-normal blood pressure until late stages of circulatory failure. Consequently, early shock may manifest only as tachycardia, prolonged capillary refill, cool extremities, or irritability, with hypotension representing a late and ominous sign. These features limit the sensitivity of standard vital-sign thresholds and underscore the need for age-adjusted clinical judgment in emergency assessment [1].

Given these challenges, considerable effort has been directed toward identifying reliable bedside indicators of pediatric shock, particularly for emergency and resource-limited settings. Classical physical examination findings—such as prolonged capillary refill time, weak pulses, cool or mottled skin, and altered mental status—remain central warning signs of impaired perfusion. Prolonged capillary refill time has been associated with a markedly increased risk of severe illness or death, although its sensitivity is limited and normal values do not exclude shock [5]. Elevated blood lactate levels represent another marker of tissue hypoperfusion, with lactate concentrations > 4 mmol/L correlating with increased mortality risk in pediatric sepsis, despite many children in shock exhibiting normal lactate values [6]. Composite measures, such as the Shock Index Pediatric Age-Adjusted (SIPA), have shown potential utility for triage but only moderate predictive performance [7]. As a result, early recognition of pediatric shock continues to rely heavily on integrated bedside clinical assessment rather than on any single diagnostic marker [1].

Despite growing awareness of these clinical features, timely identification of pediatric shock remains challenging, and current triage and screening systems have important limitations. Children in compensated shock may appear deceptively stable at initial presentation, and deterioration may occur only later during the emergency department stay. For instance, one study reported that 14% of children who ultimately developed shock showed no clear signs at triage [4]. Delays or errors in early management remain common and have been associated with adverse outcomes; a confidential inquiry in France found that over 75% of children who died from severe bacterial infections experienced suboptimal initial care [8]. While early aggressive resuscitation is widely recommended, evidence from the FEAST trial demonstrated that fluid boluses increased mortality in children with septic shock in resource-limited settings, highlighting the need for context-appropriate management strategies [9,10]. Against this background, the primary objective of the present study was to describe the distribution and clinical profile of pediatric shock within a diagnosis-based cohort of selected acute pediatric emergencies in a single-centre emergency department. The secondary objectives were to characterize the distribution of etiologic shock subtypes, to examine age-related patterns of vulnerability, and to explore associations between early bedside clinical features and the presence of shock, with a particular focus on real-world emergency care outside the intensive care unit.

## 2. Materials and Methods

### 2.1. Study Design and Setting

This retrospective observational study was conducted at a single tertiary-care hospital and was based on routinely collected clinical data from children presenting to the emergency department (ED) with acute pediatric emergencies. The study period spanned from January 2019 to December 2023. All consecutive pediatric ED presentations during the study period (N = 642) were screened using the principal diagnosis recorded as free text in ED and discharge documentation. Eligible admissions were identified using a prespecified diagnostic dictionary (diagnostic labels and keyword “trigger terms”) covering acute diarrheal syndrome (ADS), central nervous system infection (meningitis/encephalitis), decompensated heart failure, and selected acute drug intoxications (e.g., phenobarbital or diazepam) and anaphylaxis. 

All eligible admissions during this period were included consecutively. Among all screened ED presentations, 176 admissions met the prespecified eligibility criteria; after excluding records with insufficient clinical documentation for the planned analyses, 128 children remained in the analytic cohort. The remaining screened admissions (n = 466) had principal diagnoses outside the predefined emergency groups and were therefore not eligible for inclusion, irrespective of the presence or absence of shock. Shock occurring in presentations with other principal diagnoses was not subsequently excluded; such cases were outside the analytic sampling frame from the outset because eligibility was diagnosis-based. The analytic cohort was assembled from discharge summaries and detailed clinical records. These diagnostic groups were prespecified a priori as common high-acuity pediatric emergencies at our centre and were selected to capture distinct shock-relevant pathophysiological contexts (hypovolemia from diarrheal illness, CNS infection/sepsis, cardiogenic decompensation, and intoxication/anaphylaxis). This focused sampling strategy was chosen to examine shock patterns across heterogeneous emergency presentations rather than to estimate population-level prevalence. Because shock was not systematically ascertained using identical predefined clinical criteria across all 642 screened emergency department presentations, no population-level prevalence estimate was calculated. The study did not aim to include the full spectrum of pediatric emergencies. Conditions such as severe pneumonia, trauma, or hypoglycemia were not analysed because their retrospective identification based on free-text principal diagnoses was inconsistent and did not allow uniform and reproducible case ascertainment across the entire study period. The restriction to prespecified diagnostic groups was therefore intentional and aimed at ensuring internal validity and comparability of cases rather than estimating the overall burden of shock in the pediatric emergency population. A single de-identified dataset was created for analysis. Therefore, the cohort should be interpreted as a diagnosis-focused sample designed for comparative analysis of shock patterns rather than as a comprehensive representation of all pediatric emergency presentations.

### 2.2. Study Population and Case Selection

During the study period (January 2019–December 2023), a total of 642 pediatric emergency admissions were screened at the study centre. From these, 176 admissions met the predefined eligibility criteria for acute pediatric emergencies of interest, including acute diarrheal syndrome, meningitis, encephalitis, decompensated heart failure, and selected intoxications or anaphylactic reactions. Of these eligible admissions, 31 cases were excluded due to incomplete or insufficient medical documentation that did not allow reliable classification of the main diagnosis or assessment of basic demographic variables (age and sex). An additional 17 admissions were excluded because they represented repeat hospitalizations of the same patient during the study period; in such cases, only the first (index) admission was retained for analysis. Case identification followed the prespecified diagnostic dictionary and manual chart review procedure described in Section 2.1.

### 2.3. Study Population and Data Sources

The final analytic cohort comprised 128 unique children aged 0–16 years, each contributing a single index admission for an acute pediatric emergency. All included cases were identified consecutively within the predefined study period.

**Figure 1:**
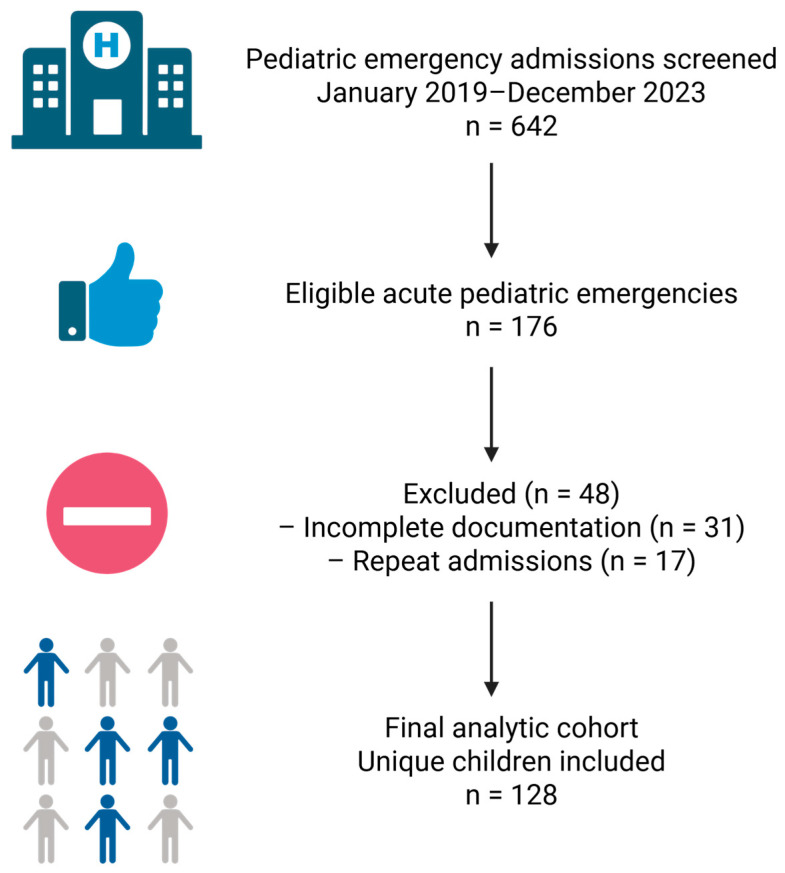
Flow diagram of patient screening, exclusion, and inclusion in the study cohort. Created with Biorender: https://app.biorender.com/illustrations/694aa0b46ab987e0eb9c5960 (accesed on 27 December 2025).

For each child, information was abstracted retrospectively from discharge letters, admission notes, daily progress notes, and nursing charts. Data were entered into a structured spreadsheet, with each row representing one patient and columns representing demographic, clinical, diagnostic, and management variables. A schematic flow diagram summarizing patient screening, exclusion, and inclusion is provided in Figure 1.

### 2.4. Variables and Data Extraction

For each child, sex (female/male) and area of residence (urban/rural) were recorded as documented in the medical record. The main diagnostic group was defined based on the principal discharge diagnosis and categorised into acute diarrheal syndrome (ADS), central nervous system infection (meningitis/encephalitis), decompensated heart failure, and intoxications/anaphylaxis. Meningitis and encephalitis were analysed together as a single CNS infection category to reduce unnecessary fragmentation and improve the stability and interpretability of between-group comparisons. Cases presenting with anaphylactic shock were classified according to their underlying trigger (most commonly drug exposure) within the intoxication category for descriptive diagnostic analyses. However, anaphylactic shock was analysed separately as a distinct etiologic subtype of pediatric shock in the shock-specific analyses. Secondary diagnoses and narrative fields were reviewed to identify relevant comorbidities and complications.

Information on length of hospital stay (LOS, in days) was reconstructed from admission and discharge dates when available. When the discharge date was missing or ambiguous, LOS was coded as missing and excluded from LOS-specific analyses. Additional variables captured from narrative notes included pre-existing cardiac disease, seizures at presentation, clinical signs of dehydration, altered mental status, and details of initial and subsequent treatment.

### 2.5. Definition and Classification of Pediatric Shock

Pediatric shock was defined retrospectively based on discharge diagnoses and detailed chart review. Children were classified as having “any shock” if either (i) the medical record explicitly documented “shock” (e.g., septic shock, anaphylactic/toxic shock, or cardiogenic shock) or equivalent terminology in Romanian; and/or (ii) the initial assessment described circulatory failure compatible with shock, including tachycardia with poor peripheral perfusion (prolonged capillary refill, cold extremities), age-specific hypotension (Pediatric Advanced Life Support thresholds: systolic blood pressure < 70 mmHg in infants; <70 + [2 × age in years] in children 1–10 years; and <90 mmHg in children ≥ 10 years), oliguria/anuria, and/or markedly altered mental status documented at presentation. Time-zero was defined as the moment of presentation to the emergency department. Therapeutic interventions (rapid IV fluid boluses and/or vasoactive support) were recorded as management variables and were considered supportive evidence when concordant with the clinical picture, but were not used as standalone criteria for shock classification. Shock classification and etiologic subtyping were performed by two pediatric physicians experienced in emergency and intensive care medicine through independent manual chart review using predefined criteria; discordant cases were adjudicated by consensus. Reviewers were not blinded to the clinical course due to the retrospective design.

Within the shock group, etiologic subtypes were assigned according to the predominant pathophysiological mechanism documented in the medical record. Children admitted with encephalitis or acute intoxications were classified as having shock only when clinical and hemodynamic features of circulatory failure were documented (e.g., tachycardia with poor peripheral perfusion, hypotension for age, or oliguria/anuria); cases with isolated central nervous system depression or drug-induced respiratory failure without circulatory compromise were not classified as shock. Hypovolemic shock was defined by severe fluid loss (e.g., profuse diarrhea, repeated vomiting, or acute bleeding) associated with signs of impaired tissue perfusion; quantitative estimation of fluid or blood volume loss was not consistently available and was not used for classification. Septic/infectious shock was defined as distributive circulatory failure in the context of documented or strongly suspected infection, supported by clinical findings and narrative documentation. Cardiogenic shock was assigned when underlying structural or functional heart disease and clinical evidence of impaired cardiac output (e.g., heart failure/low-output state) were the primary drivers of hemodynamic instability. Anaphylactic/toxic shock was considered a hemodynamic syndrome rather than a primary admission diagnostic category and was therefore classified exclusively within the shock subtype framework, irrespective of the admission diagnostic group.

To characterize management and organ support, key interventions were retrospectively identified from narrative notes and treatment summaries and coded as binary indicators (yes/no). Mechanical ventilation was coded as present if invasive ventilation via endotracheal tube or tracheostomy was documented during the index admission. Vasopressor/inotropic support was coded when continuous infusion of vasoactive agents (e.g., noradrenaline, dopamine, adrenaline) was documented. Intensive care unit (ICU) transfer/admission was recorded when the child was documented as being monitored in a dedicated intensive care area (“Terapie Intensivă” or equivalent). Cardiorespiratory arrest was identified by documentation of “stop cardiorespirator” and/or cardiopulmonary resuscitation. These variables were used descriptively to characterize the burden of organ support across shock subtypes.

Clinically relevant co-morbidities and admission features were derived as potential early markers of shock. Pre-existing cardiac disease was coded as present when structural or functional heart disease (congenital heart defect, cardiomyopathy, chronic heart failure) was documented in admission or past history. Seizures at presentation were coded if admission notes described convulsions, tonic–clonic seizures, or status epilepticus. Severe dehydration was recorded when examination documented major fluid depletion (e.g., decreased skin turgor, dry mucous membranes, sunken eyes/fontanelle, oliguria/anuria). Altered mental status/coma was defined by impaired consciousness at presentation (e.g., somnolence, lethargy, stupor, or coma, including “stare generală gravă/alterată” in Romanian documentation). Initial management variables (intravenous fluid therapy, early intravenous antibiotics, and oxygen therapy) were extracted from narrative treatment fields and coded as binary indicators (yes/no).

### 2.6. Age and Length-of-Stay Measures

Age at admission was analyzed as both a continuous variable (years, including decimals derived from years and months; infants < 1 year expressed as fractions of a year) and as a categorical variable using four pediatric strata: <1 year, 1–4 years, 5–9 years, and 10–16 years. Length of stay (LOS) was calculated in days as the difference between discharge and admission dates.

### 2.7. Statistical Analysis

The initial dataset was compiled, coded, cleaned, and verified in Microsoft Excel 365 (Microsoft Corp., Redmond, WA, USA), and all statistical analyses were conducted using IBM SPSS Statistics, version 26 (IBM Corp., Armonk, NY, USA).

Categorical variables are reported as counts and percentages using non-missing values as denominators; effective denominators are indicated in the tables where applicable. Continuous variables (age and length of stay) are summarized using mean ± standard deviation (SD) and median with interquartile range (IQR). Length-of-stay (LOS) distributions were right-skewed and influenced by a small number of prolonged admissions; therefore, both measures are reported. LOS could not be reconstructed for all patients; in particular, one septic/infectious shock case had incomplete admission/discharge date documentation.

Inferential analyses were performed in several steps. First, demographic characteristics (age, sex, area of residence) and resource use (LOS) were compared across the four main diagnostic groups (acute diarrheal syndrome, CNS infection [meningitis/encephalitis], heart failure, and intoxications). Given the non-normal distribution of age and LOS and the small sample sizes within some diagnostic categories, between-group differences for continuous variables were assessed using the Kruskal–Wallis H test. Associations between diagnostic category and categorical variables were evaluated using Pearson’s χ^2^ test or Fisher’s exact test, as appropriate.

Second, the cohort was stratified by shock status (“any shock” vs. “no shock”). Age and LOS were compared between groups using the Mann–Whitney U test, while categorical variables (sex, area of residence, and diagnostic group) were compared using Pearson’s χ^2^ test or Fisher’s exact test, as appropriate.

Third, analyses were restricted to children with shock to characterize etiologic subtypes (anaphylactic/toxic, hypovolemic, septic/infectious, and cardiogenic). Age, sex, area of residence, and LOS were compared across subtypes using the Kruskal–Wallis H test for continuous variables and Pearson’s χ^2^ test or Fisher’s exact test for categorical variables, as appropriate.

Finally, baseline clinical features associated with shock status were explored. Age was examined both as a continuous variable and after reclassification into four pediatric age strata (<1 year, 1–4 years, 5–9 years, and 10–16 years). Associations between shock status and age strata, pre-existing cardiac disease, seizures at presentation, severe dehydration, and altered mental status or coma were assessed using Pearson’s χ^2^ test or Fisher’s exact test, depending on expected cell counts. Early management variables (intravenous fluids, early intravenous antibiotics, oxygen therapy, ICU transfer, and organ support) were summarized descriptively only, because treatment decisions may reflect clinical recognition of shock and may therefore introduce incorporation bias if treated as independent predictors.

All inferential tests were two-sided, and a *p*-value < 0.05 was considered statistically significant. Given the exploratory nature of the study and the limited sample size, no formal adjustment for multiple comparisons was applied, and *p*-values are interpreted as indicators of association rather than definitive evidence of causality. Missing data were handled using complete-case analysis for each comparison, and effective denominators are reported in the corresponding tables.

## 3. Results

### 3.1. Overall Cohort Characteristics

The study included 128 children with acute pediatric emergencies. The mean age of the cohort was 5.4 ± 4.6 years, with a median of 3.7 years (IQR 2.5–8.0) and a range from 0.03 years (~11 days) to 16 years. In total, 36 of 128 children (28.1%) fulfilled criteria for pediatric shock, whereas 92 presented with acute emergencies without shock. Overall in-hospital mortality was 4.7% (6/128); all deaths occurred among children with shock (16.7%, 6/36), whereas no deaths were recorded among children without shock (0/92). The overall demographic and clinical characteristics of the cohort are summarised in Table 1.

Among the study population, 76 children (59.4%) were male and 52 (40.6%) were female. Most patients originated from rural areas: 76 (59.4%) lived in rural settings and 52 (40.6%) in urban areas. The main diagnostic categories at admission were acute diarrheal syndrome (ADS) in 50 children (39.1%), intoxications with phenobarbital or diazepam and/or documented anaphylaxis in 42 (32.8%), decompensated heart failure in 19 (14.8%), and central nervous system infection (meningitis/encephalitis) in 17 (13.3%).

Length of stay (LOS) was available for 93/128 children; the median LOS was 8 days (IQR 7–11) and the mean LOS was 9.9 ± 8.9 days (range 2–74) (Table 1).

Inter-group differences in age, sex, area of residence, and LOS by diagnostic category are summarized in Table 2; subsequent analyses examine shock status and etiologic subtypes in detail.

### 3.2. Diagnostic Patterns and Resource Use Across the Cohort

#### 3.2.1. Sex and Area of Residence by Diagnostic Category

When stratified by the main diagnostic group (ADS, CNS infection, heart failure, intoxications), the distribution of sex differed significantly across diagnoses (χ^2^(3) = 8.10, *p* = 0.044). CNS infection and heart failure were predominantly diagnosed in boys, whereas ADS showed a nearly balanced sex distribution and intoxications had a moderate male predominance (Table 2).

Area of residence did not differ significantly across diagnostic categories (χ^2^(3) = 2.14, *p* = 0.544; Table 2), although rural origin was numerically more frequent in heart failure and CNS infection.

#### 3.2.2. Age Differences Between Diagnostic Groups

Age differed markedly across the four main diagnostic categories; the global comparison showed a highly significant difference in age between groups (Kruskal–Wallis H(3) = 45.0, *p* < 0.001) (Table 2).

Intoxications involved older children, whereas ADS and heart failure clustered in younger ages; CNS infection showed intermediate ages (Table 2).

#### 3.2.3. Length of Stay by Diagnostic Category

Length of stay (LOS) could be reconstructed for 93 of the 128 children, and LOS varied significantly across diagnostic groups (Kruskal–Wallis H(3) = 17.2, *p* < 0.001). These comparisons are based on patients with available LOS data. Among children with acute diarrheal syndrome (ADS; n = 50 with LOS data), the mean LOS was 8.4 ± 2.6 days, with a median of 8.5 days (IQR 7–10; range 2–14). For intoxications/anaphylaxis (n = 14 with LOS data), LOS was shorter, with a mean of 5.7 ± 2.2 days and a median of 6.0 days (IQR 5–6.8; range 2–11). Children with CNS infection (meningitis/encephalitis; n = 14 with LOS data) had a mean LOS of 10.2 ± 6.9 days and a median of 10.0 days (IQR 5.0–12.8; range 3–30). The longest stays were observed in children with heart failure (n = 15 with LOS data), who had a mean LOS of 18.6 ± 18.3 days and a median of 13.0 days (IQR 8–18; range 3–74). Overall, LOS was shortest in intoxications/anaphylaxis and longest in heart failure; prolonged admissions (>30 days) occurred only in heart failure and CNS infection.

Given these marked differences in age and resource use between diagnostic categories, the presence of pediatric shock was next examined as a further stratifying factor in this heterogeneous cohort.

### 3.3. Children with and Without Shock

When stratified by shock status, 36 children (28.1%) fulfilled criteria for any shock and 92 (71.9%) had no documented shock. Children with shock were younger (median 1.2 vs. 3.7 years; *p* = 0.003) and showed a non-significant trend toward longer LOS (median 10 vs. 8 days; *p* = 0.063). These LOS comparisons are based on patients with available data, as length of stay could not be reconstructed for all anaphylactic/toxic shock cases. Sex and area of residence did not differ between groups (both *p* = 0.211). The distribution of diagnostic groups differed (*p* < 0.001), with heart failure disproportionately represented among shock cases (Table 3).

### 3.4. Etiologic Subtypes of Pediatric Shock

Among the 36 children with pediatric shock, etiologic classification yielded four subtypes: anaphylactic/toxic (n = 10, 27.8%), hypovolemic (n = 8, 22.2%), septic/infectious (n = 5, 13.9%), and cardiogenic (n = 13, 36.1%) (Table 4). Age distribution differed significantly across subtypes (*p* = 0.007, Kruskal–Wallis test), with anaphylactic/toxic shock occurring in older children and hypovolemic/septic shock clustering in infancy. Sex and area of residence did not differ significantly across shock subtypes (*p* = 0.65 and *p* = 0.90, respectively). Length of stay could not be reliably reconstructed for anaphylactic shock; among the remaining subtypes, LOS differed (*p* = 0.025), with the shortest stays in hypovolemic shock and the longest in septic and cardiogenic shock (Table 4).

### 3.5. Clinical Management and Organ Support in Shock

Patterns of organ support differed across shock subtypes (Table 5). No children with anaphylactic/toxic shock required mechanical ventilation, vasopressor therapy, or intensive care admission, consistent with rapid clinical stabilization after anti-anaphylactic treatment. Septic/infectious shock showed the highest intensity of support, with ICU management in 4/5 cases (80.0%) and need for mechanical ventilation and vasopressors in 1/5 (20.0%). Cardiogenic shock required ICU transfer/admission in 5/13 cases (38.5%), without documented mechanical ventilation or vasopressor use. In hypovolemic shock, ICU admission occurred in 1/8 (12.5%), and cardiorespiratory arrest was documented in 1/8 (12.5%). In-hospital deaths occurred predominantly in hypovolemic shock (4/8, 50.0%), with one death in septic/infectious shock (1/5, 20.0%) and one death in cardiogenic shock (1/13, 7.7%); no deaths were recorded in anaphylactic/toxic shock (0/10).

### 3.6. Age Strata, Comorbid Conditions, and Baseline Clinical Features According to Shock Status

Shock Was Most Frequent in Infants < 1 Year, but a Second Notable Cluster Was Observed Among Children Aged 5–9 Years (Table 6).

Pre-existing cardiac disease, severe dehydration, and altered mental status/coma were more frequent in children with shock than in those without (*p* = 0.002, *p* < 0.001, and *p* = 0.018, respectively), whereas seizures at presentation did not differ significantly (Table 7).

The data presented in Table 8 are reported descriptively and were intentionally excluded from inferential statistical analyses to prevent incorporation bias.

## 4. Discussion

The present study [11,12,13] provides a real-world emergency department perspective on pediatric shock across a heterogeneous spectrum of acute conditions outside the intensive care setting. By integrating multiple emergency diagnoses rather than a single etiology, it shows that shock frequently complicates diverse presentations and may be initially under-recognised. This approach complements the predominantly ICU-based literature and highlights age-related vulnerability patterns together with simple, readily identifiable bedside clinical indicators relevant for early recognition [11,12,13,14,15]. Beyond confirming known patterns of pediatric shock, this study adds several novel perspectives. First, it provides an emergency department-based, age-integrated analysis of shock across multiple acute conditions. Second, the age-stratified approach identified a bimodal vulnerability pattern, with shock clustering not only in infants but also in school-aged children (5–9 years), a group less commonly highlighted in pediatric shock patterns in larger cohorts. Third, simple bedside features—such as pre-existing cardiac disease, severe dehydration, and altered mental status—were more frequent among children with shock across diverse etiologies, suggesting their potential value as pragmatic early clinical indicators in settings where advanced diagnostics may be limited. Age distributions and diagnostic patterns in our cohort are broadly concordant with international data. Children with shock were substantially younger than those without shock, and age-stratified analyses showed that shock clustered in infants < 1 year and in children aged 5–9 years, whereas toddlers and adolescents were less frequently affected. Large epidemiological studies and reviews of pediatric sepsis consistently report the highest incidence and mortality of septic shock in infants and young children, particularly in low- and middle-income settings [12,13,14]. In parallel, the prominent contribution of acute diarrhoeal disease and severe dehydration to hypovolemic shock in our series is aligned with global data indicating that diarrhoeal disease remains one of the leading causes of morbidity and mortality in young children worldwide and a major precipitant of hypovolemic collapse [15,16,17,18]. Likewise, the contribution of central nervous system infection (meningitis/encephalitis) to shock episodes in our cohort is consistent with reports that severe sepsis and septic shock in children frequently arise from invasive bacterial infections of the respiratory and central nervous systems [11,12,13,14].

When the 36 shock episodes were examined by etiologic subtype, the cohort displayed marked heterogeneity, encompassing anaphylactic/toxic, hypovolemic, septic/infectious, and cardiogenic shock, with distinct age profiles and lengths of stay. This mirrors prior descriptions of pediatric shock in emergency and critical care settings, where sepsis and hypovolemia typically account for the bulk of cases, with cardiogenic and anaphylactic shock representing smaller but clinically important fractions [14,19,20,21,22,23]. In our cohort, cardiogenic shock occurred predominantly in children with pre-existing heart failure, which is consistent with previous studies identifying decompensated congenital or acquired heart disease as the main substrate for pediatric cardiogenic shock and as a determinant of prolonged hospitalization and increased mortality compared with previously healthy peers [14,20,21,22,23]. Beyond etiologic labels, several simple bedside features were strongly associated with shock and are well supported by the literature. Pre-existing cardiac disease, severe dehydration, and altered mental status or coma at admission were markedly more frequent among children with shock than among those without shock, highlighting their potential value as pragmatic early clinical indicators. The strong association between severe dehydration and shock dovetails with the global burden of dehydrating diarrhoea as a leading cause of hypovolemic collapse in infants and young children [16,17,18]. Finally, the enrichment of altered mental status in the shock group is consistent with sepsis guidelines and clinical marker studies that emphasise impaired consciousness as a key red-flag sign of severe illness and septic shock in children [24,25,26]. Seizures at presentation were relatively uncommon and did not clearly discriminate between groups in our cohort, which may relate both to limited power and to the fact that seizures can accompany a variety of infectious and non-infectious emergencies independent of hemodynamic status. From a practical standpoint, these factors represent simple bedside features that can be identified early in the emergency department and may support earlier monitoring, escalation of care, and timely referral to higher-acuity settings.

Analyses of early management patterns showed that intravenous fluid therapy and empirical intravenous antibiotics were widely used across the cohort, with broadly similar frequencies in children with and without shock, whereas oxygen therapy was rarely documented. This pattern is not at odds with current guideline-based practice, which recommends early fluid resuscitation and prompt antimicrobial therapy for a broad range of suspected septic and pre-shock states in children, even before overt shock is fully established [27,28]. In other words, our data suggest that basic resuscitative measures are initiated at similarly low thresholds for all severely ill children in this emergency department, and that the development of overt shock is driven more by underlying pathophysiology and initial severity than by major differences in early treatment intensity. Taken together, the present findings are broadly congruent with international experience: in this selected cohort of acute pediatric emergencies, shock was relatively common, etiologically heterogeneous, and concentrated in specific age bands and diagnostic categories, with a small number of readily recognisable bedside clinical features that may assist early identification of more severely ill children [11,12,13,14,15,19,24,25,26,27,28,29].

Several limitations should be acknowledged when interpreting these findings. First, the study used a retrospective, single-centre design based on routinely collected data from discharge summaries and narrative clinical notes. As a result, case ascertainment and variable coding depended on the completeness and clarity of existing documentation rather than on prospectively standardised forms. Misclassification of both shock status and shock subtype cannot be excluded, particularly in children with mixed clinical pictures or in whom hemodynamic parameters were incompletely recorded. Furthermore, because shock was not captured prospectively using a standardized hemodynamic protocol, the timing of onset and the exact severity of circulatory failure could not be uniformly assessed across all cases.

Second, the sample size was modest, and the distribution of children across diagnostic categories and shock subtypes was uneven. Some strata contained very small numbers, which limits the precision of estimates and precludes robust conclusions regarding differences between individual subtypes, especially for outcomes such as length of stay, need for organ support, or in-hospital death. Differences in local intensive-care organisation may limit direct comparability with stand-alone PICUs in other healthcare systems. The analyses of comorbidities and early clinical features were similarly constrained by low event counts in certain cells, necessitating reliance on χ^2^ or Fisher’s exact tests without further multivariable adjustment. As specified in the statistical analysis, no correction for multiple comparisons was applied, and the *p*-values reported should therefore be interpreted as exploratory signals of association rather than definitive evidence of causality.

Third, the dataset was restricted to a focused set of acute pediatric emergencies—acute diarrhoeal syndrome, central nervous system infection, heart failure, and intoxications—which may not capture the full spectrum of conditions associated with shock in other hospitals or regions. Length of stay could be reliably reconstructed only for a subset of children, most notably in anaphylactic shock after inter-hospital transfer; this non-random missingness may have influenced LOS comparisons. In addition, detailed hemodynamic monitoring data, standardized severity scores, and long-term follow-up outcomes were not available, which limits generalisability and prevents assessment of longer-term consequences. Despite these constraints, the study provides a granular real-world snapshot of pediatric shock across a heterogeneous group of acute emergencies and highlights several clinically recognisable patterns that may help guide future prospective research. From a practical and clinical perspective, the present findings underscore that shock should be actively considered across a wide range of acute presentations, not only in classic septic scenarios. The clustering of cases in infants and in children aged 5–9 years suggests the need for heightened clinical vigilance in these age groups, even when initial vital signs appear relatively preserved. Simple bedside information—particularly cardiac comorbidity, dehydration, and altered consciousness—may support the early recognition of circulatory failure and facilitate timely monitoring and escalation of care.

## 5. Conclusions

Within this diagnosis-based single-centre emergency cohort, pediatric shock emerged as a frequent and etiologically heterogeneous complication of selected acute pediatric emergencies, extending beyond classic septic presentations. Shock clustered in specific age groups—particularly infants and children aged 5–9 years—and was consistently accompanied by readily identifiable bedside features such as pre-existing cardiac disease, severe dehydration, and altered mental status. These findings underscore the importance of early, clinically driven assessment in pediatric emergency settings. Simple bedside information available at presentation may support the early recognition of circulatory failure, facilitate closer monitoring and timely escalation of care, and thereby contribute to improved clinical vigilance outside the intensive care unit.

## Figures and Tables

**Table 1:** Overall characteristics of the pediatric emergency cohort (N = 128).

Parameter	Characteristic	Value
Age (years)	Mean ± SD	5.4 ± 4.6
	Median (IQR)	3.7 (2.5–8.0)
	Range	0.03–16.0
Sex n (%)	Female	52 (40.6%)
	Male	76 (59.4%)
Area of residence n (%)	Rural	76 (59.4%)
	Urban	52 (40.6%)
Main diagnostic group n (%)	Acute diarrheal syndrome (ADS)	50 (39.1%)
	Intoxications *	42 (32.8%)
	Heart failure	19 (14.8%)
	Central nervous system infection	17 (13.0%)
Length of stay days **	Mean ± SD	9.9 ± 8.9
	Median (IQR)	8 (7–11)
	Range	2–74

* Intoxications include admissions for fenobarbital or diazepam exposure. Cases complicated by anaphylactic shock are included here based on the underlying trigger, while anaphylactic shock is analysed separately as a shock subtype. ** Length of stay available for 93/128 children.

**Table 2:** Age, sex, area of residence, and length of stay according to main diagnostic group.

Variable	ADS (n = 50)	CNS Infection (n = 17)	Heart Failure (n = 19)	Intoxications (n = 42)	*p*-Value
**Age (years)**					
Mean ± SD	2.6 ± 1.2	5.7 ± 3.8	4.0 ± 4.7	9.1 ± 4.8	<0.001 *
Median (IQR)	3.0 (1.6–3.7)	6.0 (2.6–8.1)	1.0 (0.5–7.5)	11.4 (4.0–13.0)	<0.001 *
**Sex**					
Female, n (%)	26 (52.0%)	3 (17.6%)	5 (26.3%)	18 (42.9%)	0.044 **
Male, n (%)	24 (48.0%)	14 (82.4%)	14 (73.7%)	24 (57.1%)	0.044 **
**Area of residence**					
Rural, n (%)	28 (56.0%)	12 (70.6%)	13 (68.4%)	23 (54.8%)	0.544 **
Urban, n (%)	22 (44.0%)	5 (29.4%)	6 (31.6%)	19 (45.2%)	0.544 **
**Length of stay (days)**					
Mean ± SD	8.4 ± 2.6	10.2 ± 6.9	18.6 ± 18.3	5.7 ± 2.2	<0.001 *
Median (IQR)	8.5 (7–10)	10.0 (5.0–12.8)	13 (8–18)	6.0 (5–6.8)	<0.001 *

* Kruskal–Wallis test; ** Chi-square test; bold values denote statistical significance. Abbreviations: ADS, acute diarrheal syndrome; CNS, central nervous system; IQR, interquartile range; SD, standard deviation.

**Table 3:** Comparison of demographic and clinical characteristics between children with and without pediatric shock.

Variable	No Shock (n = 92), n (%)	Any Shock (n = 36), n (%)	*p*-Value	OR (95% CI)
**Age (years)**				
Mean ± SD	6.0 ± 4.6	3.8 ± 4.2	0.003 *	
Median (IQR)	3.7 (2.9–10.0)	1.2 (0.7–6.0)		
**Sex, n (%)**			0.211 **	
Female	41 (44.6%)	11 (30.6%)		1.00 (reference)
Male	51 (55.4%)	25 (69.4%)		1.83 (0.81–4.14)
**Area of residence, n (%)**			0.211 **	
Rural	51 (55.4%)	25 (69.4%)		1.00 (reference)
Urban	41 (44.6%)	11 (30.6%)		0.55 (0.24–1.24)
**Main diagnostic group, n (%)**			<0.001 **	
Intoxications #	32 (34.8%)	10 (27.8%)		1.00 (reference)
Acute diarrheal syndrome (ADS)	42 (45.7%)	8 (22.2%)		0.61 (0.23–1.64)
CNS infection (meningitis/encephalitis)	12 (13.0%)	5 (13.9%)		1.33 (0.38–4.65)
Heart failure	6 (6.5%)	13 (36.1%)		6.93 (2.05–23.4)
**Length of stay, days ##**				
Mean ± SD	8.4 ± 4.0	14.6 ± 15.7	0.063 *	
Median (IQR)	8.0 (7.0–10.0)	10.0 (7.0–15.0)		

# Intoxications with fenobarbital, diazepam, or documented anaphylactic shock. ## Length of stay available for 93 children (no shock: n = 70; any shock: n = 23). * *p*-value from Mann–Whitney U test (continuous variables). ** *p*-value from Pearson’s χ^2^ test (categorical variables, including the distribution of main diagnostic group). Bold values denote statistical significance. OR, odds ratio; CI, confidence interval. Odds ratios are from univariable comparisons; female sex, rural residence, and intoxications were used as reference categories. LOS analyses were performed on patients with available data. Abbreviations: ADS, acute diarrheal syndrome; CNS, central nervous system; LOS, length of stay; IQR, interquartile range; SD, standard deviation.

**Table 4:** Demographic and clinical characteristics according to pediatric shock subtype (n = 36).

Variable	Anaphylactic (n = 10)	Hypovolemic (n = 8)	Septic (n = 5)	Cardiogenic (n = 13)
**Age, years**				
Mean ± SD	7.4 ± 3.9	1.1 ± 0.1	5.0 ± 4.4	5.3 ± 5.0
Median (IQR)	6.0 (5.0–10.0)	1.1 (1.1–1.1)	4.5 (1.3– 6.0)	4.8 (1.0–7.5)
**Sex, n (%)**				
Female	4 (40.0%)	4 (50.0%)	0 (0.0%)	3 (23.1%)
Male	6 (60.0%)	4 (50.0%)	5 (100.0%)	10 (76.9%)
**Area of residence, n (%)**				
Rural	6 (60.0%)	6 (75.0%)	4 (80.0%)	9 (69.2%)
Urban	4 (40.0%)	2 (25.0%)	1 (20.0%)	4 (30.8%)
Length of stay, days *				
Mean ± SD	4.4 ± 0.5	5.9 ± 3.2	15.0 ± 10.6	18.8 ± 18.9
Median (IQR)	4.0 (4.0–5.0)	6.5 (2.8–8.2)	12.5 (10.2–17.2)	13.0 (8.0–16.0)
**In-hospital death, n (%)**	0 (0.0%)	4 (50.0%)	1 (20.0%)	1 (7.7%)

Note: * Length of stay was reconstructed from recorded admission/discharge dates; Bold values denote statistical significance; one septic/infectious case had incomplete date documentation. Global comparisons across shock subtypes: age *p* = 0.007 (Kruskal–Wallis test); length of stay *p* = 0.025 among non-anaphylactic subtypes (Kruskal–Wallis); sex *p* = 0.65 and area of residence *p* = 0.90 (Chi-square tests).

**Table 5:** Organ support and intensive-care management according to shock subtype.

Variable	Anaphylactic (n = 10)	Hypovolemic (n = 8)	Septic (n = 5)	Cardiogenic (n = 13)
Mechanical ventilation, n (%)	0 (0.0%)	0 (0.0%)	1 (20.0%)	0 (0.0%)
Vasopressor support, n (%)	0 (0.0%)	0 (0.0%)	1 (20.0%)	0 (0.0%)
ICU transfer/admission, n (%)	0 (0.0%)	1 (12.5%)	4 (80.0%)	5 (38.5%)
Cardiorespiratory arrest, n (%)	0 (0.0%)	1 (12.5%)	0 (0.0%)	0 (0.0%)

Note: Organ-support variables were extracted from structured clinical documentation and coded as binary (yes/no). Percentages are calculated within each shock subtype using the total number of cases in that subtype as denominator. Variables are not mutually exclusive; a single patient may contribute to more than one category.

**Table 6:** Age classes according to the presence of pediatric shock.

Variable	No Shock (n = 92), n (%)	Any Shock (n = 36), n (%)	*p*-Value
<1 year	6 (6.5%)	12 (33.3%)	<0.001 *
1–4 years	53 (57.6%)	11 (30.6%)	
5–9 years	8 (8.7%)	9 (25.0%)	
10–16 years	25 (27.2%)	4 (11.1%)	

* Chi-square test. Bold values denote statistical significance.

**Table 7:** Clinical co-morbidities and admission features associated with pediatric shock.

Variable	No Shock (n = 92), n (%)	Any Shock (n = 36), n (%)	*p*-Value *
Pre-existing cardiac disease	6 (6.5%)	10 (27.8%)	**0.002**
Seizures at presentation	2 (2.2%)	2 (5.6%)	0.310
Severe dehydration	4 (4.3%)	10 (27.8%)	**<0.001**
Altered mental status/coma	11 (12.0%)	11 (30.6%)	**0.018**

* Fisher’s exact test. Bold values denote statistical significance.

**Table 8:** Initial management patterns in children with and without pediatric shock.

Variable	No Shock (n = 92), n (%)	Any Shock (n = 36), n (%)
Intravenous fluid therapy	50 (54.3%)	16 (44.4%)
Early intravenous antibiotics	12 (13.0%)	7 (19.4%)
Oxygen therapy	0 (0.0%)	1 (2.8%)

Early management variables are summarized descriptively (Table 8) and were not subjected to hypothesis testing to avoid incorporation bias.

## Data Availability

Data are contained within the article.
